# Structured mentorship program for the ABR international medical graduates alternate pathway for medical physicists in diagnostic imaging

**DOI:** 10.1002/acm2.13166

**Published:** 2021-01-09

**Authors:** Francesco Ria, Joshua M. Wilson, Jeffrey Nelson, Ehsan Samei

**Affiliations:** ^1^ Carl E. Ravin Advanced Imaging Labs and Clinical Imaging Physics Group Duke University Health System Durham NC USA; ^2^ Clinical Imaging Physics Group and Medical Physics Graduate Program Duke University Health System Durham NC USA; ^3^ Clinical Imaging Physics Group Duke University Health System Durham NC USA; ^4^ Carl E. Ravin Advanced Imaging Labs Clinical Imaging Physics Group Medical Physics Graduate Program Departments of Radiology, Physics, Biomedical Engineering, and Electrical and Computer Engineering Duke University Durham NC USA

Dear editor,

According to the US Census Bureau, recent immigrants to the United States are more likely to have a college education than earlier immigrants or the native born.^1^ In particular, in 2018 13.9% of immigrants ages 25 and older hold a postgraduate degree^2^ compared to about 13.1% of US adults.^1^ However, in a highly technological discipline like medical physics, even the highest education needs to be integrated with specific competencies sufficient to practice imaging physics independently.^3^ To this end, the American Board of Radiology (ABR) provides an alternate pathway to board certification for medical physicists trained in countries other than the United States and Canada.^4^

To be considered as a candidate for the Alternate Pathway, an applicant must meet several requirements, including completing a Structured Mentorship Program (SMP). The SMP is^4^

1. conducted through a sponsoring department at an institution that has a residency program accredited through the Commission on Accreditation of Medical Physics Education Programs (CAMPEP),
2. under a supervising medical physicist who is a diplomate of the ABR,
3. for a minimum of 3 years in the same institution.

The ABR lists six high‐level competencies that must be met along with minimum SMP portfolio activities. These competencies are not as granularly defined as those of CAMPEP’s standards for residency programs. Thus, implementing an SMP allows for flexibility in tailoring the Training Plan to the candidate, which can also be a daunting undertaking.

At Duke University, we planned a 3 yr SMP that was approved by the ABR Medical Physics Credentials Evaluation Committee in May 2017. The candidate completed the SMP, on schedule, in 2020. In this letter we would like to share our experience to provide useful information for other institutions and international graduates who want to follow the same pathway.

## 1 SMP GOAL AND STRUCTURE

The objective of the SMP at Duke University Medical Center was to provide a mentorship program for medical physicists trained in foreign countries to meet the requirements of the ABR and become board certified in Diagnostic Physics. Duke conducts an Imaging Physics Residency Program that is accredited through the CAMPEP. The SMP candidate maintained a level of apprenticeship comparable to that of residents in the CAMPEP Residency Program to ensure the SMP candidate meets the minimum level of competence sufficient to perform all aspects of routine diagnostic imaging tasks. Additional flexibility was considered in areas where the candidate had a strong demonstrated proficiency. An overall objective was maintained for the candidate to contribute to the oversight of safe and accurate imaging procedures. In addition, the SMP provided an environment for the candidate to demonstrate competent performance in other aspects of an imaging physicist’s responsibilities such as teaching, research, radiation safety, and administration.

The SMP was closely associated with both the Imaging Physics Residency Program and the Medical Physics Graduate Program, which together offer an extensive array of basic and advanced medical physics graduate courses and practical training for a candidate. A candidate’s progress was managed by a Facility Supervisor (the Residency Program Director), assisted by the Imaging Physics Residency Program’s Assistant Director, as well as several rotation mentors. All clinical rotation mentors were members of the Duke Clinical Imaging Physics Group (CIPG, http://cipg.duhs.duke.edu), who provide clinical physics support for the imaging operations across the Duke University Health System. The SMP candidate had access to clinical resources and testing equipment through the CIPG. Staff of the Radiation Safety Division in the Duke Occupational & Environmental Safety Office were also involved in the mentorship program to provide rotational mentor support and track the candidates’ radiation safety training and occupational dosimetry badge reports.

## 2 SMP REQUIREMENTS AND COMPETENCIES

The duration of the SMP totaled 36 consecutive months. Following the ABR guidelines, the mentorship included oversight in both clinical and technical areas of imaging physics for imaging modalities: radiography, fluoroscopy and interventional radiology, mammography, computed tomography, ultrasound, and magnetic resonance. All modalities included routine competency requirements of physics fundamentals, instrumentation and system operation, regulatory requirements, image quality attributes and parameters, and QC and QA program evaluation. Additionally, each modality had a unique set of competencies, listed below.

### 2.1 Radiography

Radiation safety; Equipment evaluation including collimation accuracy, beam quality assessment, automatic exposure control performance, phantom image quality evaluation, and computed radiography image reader performance; Dosimetry measurements including exposure reproducibility/linearity and patient entrance skin exposure.

### 2.2 Fluoroscopy and interventional radiology

Radiation safety; Equipment evaluation including collimation, resolution, beam quality assessment, automatic exposure control performance, and phantom image quality evaluation; Dosimetry including measuring exposure rates and evaluating patient entrance skin dose exposure.

### 2.3 Mammography

Performing medical physicist testing following MQSA/ACR requirements including collimation, artifact evaluation, detector uniformity, system resolution, and SNR/CNR measurements; Dosimetry including measuring and evaluating breast entrance dose, average glandular dose, and automatic exposure control performance.

### 2.4 Computed tomography

Radiation safety; Dosimetry principles, including measuring CT dose index; Equipment evaluation including slice thickness accuracy, CT number accuracy, uniformity, and high/low contrast resolution.

### 2.5 Ultrasound

Equipment evaluation including resolution, uniformity, depth of penetration, and Doppler/color‐flow evaluation.

### 2.6 Magnetic resonance imaging

MRI safety; Equipment evaluation including phase stability, magnetic field homogeneity, radio frequency calibration, SNR, intensity uniformity, MR spectroscopy water and metabolic peak areas, and volume‐of‐interest accuracy.

## 3 SMP CANDIDATE PROGRESS EVALUATION

For each modality rotation, the candidate reported a list of completed competencies for review and evaluation by the Facility Supervisor and rotation mentor. The candidate also maintained rotation portfolios for each module including:

- List of competencies the candidate had mastered;
- List of the physics responsibilities carried out during the rotation;
- List of completed equipment evaluations performed;
- Summary of additional activities, including operational improvements and clinically oriented projects;
- Training resources and references used for the module;
- Evaluations of the candidate by rotation mentor and Facility Supervisor.

The candidate completed the six clinical rotations, performed 51 equipment evaluations, and three clinical quality improvement projects, which led to four conference presentations and two peer‐reviewed publications.^5^, ^6^ Furthermore, the candidate passed ABR Part‐1 exam in 2018. Throughout the mentorship, bi‐monthly, recurring individual meetings were scheduled between the candidate and the Facility Supervisor to review SMP progress, including rotation competencies and projects.

## 4 CONCLUSION OF CANDIDATE’S SMP

The 3‐yr SMP was successfully completed as planned and on‐schedule. The international graduate, who was qualified as a medical physicist in his country of origin and had practiced there, reviewed and demonstrated clinical competency of diagnostic imaging modalities, techniques, and procedures. The ABR approved the proposed SMP and admitted the candidate to Part‐2 exam. The ABR International Medical Graduates Alternate Pathway provided the candidate specific competencies to practice diagnostic imaging physics independently offering alongside opportunities to improve in both clinical and research activities.
